# Comparison of coenzyme Q10 or fish oil for prevention of intermittent hypoxia-induced oxidative injury in neonatal rat lungs

**DOI:** 10.1186/s12931-021-01786-w

**Published:** 2021-07-05

**Authors:** Christina D’Agrosa, Charles L. Cai, Faisal Siddiqui, Karen Deslouches, Stephen Wadowski, Jacob V. Aranda, Kay D. Beharry

**Affiliations:** 1 Department of Pediatrics and Ophthalmology, Division of Neonatal-Perinatal Medicine Clinical and Translational Research Labs, State University of New York, Downstate Medical Center, 450 Clarkson Avenue, Box 49, Brooklyn, NY 11203 USA; 2 Department of Ophthalmology, State University of New York, Downstate Medical Center, Brooklyn, NY 11203 USA; 3 State University of New York Eye Institute, New York, NY USA

**Keywords:** Coenzyme Q10, Fish oil, Intermittent hypoxia, Lung injury, Oxidative stress

## Abstract

**Background:**

Neonatal intermittent hypoxia (IH) results in oxidative distress in preterm infants with immature antioxidant systems, contributing to lung injury. Coenzyme Q10 (CoQ10) and fish oil protect against oxidative injury. We tested the hypothesis that CoQ10 is more effective than fish oil for prevention of IH-induced lung injury in neonatal rats.

**Methods:**

Newborn rats were exposed to two clinically relevant IH paradigms at birth (P0): (1) 50% O_2_ with brief hypoxia (12% O_2_); or (2) room air (RA) with brief hypoxia (12% O_2_), until P14 during which they were supplemented with daily oral CoQ10, fish oil, or olive oil from P0 to P14. Pups were studied at P14 or placed in RA until P21 with no further treatment. Lungs were assessed for histopathology and morphometry; biomarkers of oxidative stress and lipid peroxidation; and antioxidants.

**Results:**

Of the two neonatal IH paradigms 21%/12% O_2_ IH resulted in the most severe outcomes, evidenced by histopathology and morphometry. CoQ10 was effective for preserving lung architecture and reduction of IH-induced oxidative stress biomarkers. In contrast, fish oil resulted in significant adverse outcomes including oversimplified alveoli, hemorrhage, reduced secondary crest formation and thickened septae. This was associated with elevated oxidants and antioxidants activities.

**Conclusions:**

Data suggest that higher FiO_2_ may be needed between IH episodes to curtail the damaging effects of IH, and to provide the lungs with necessary respite. The negative outcomes with fish oil supplementation suggest oxidative stress-induced lipid peroxidation.

**Supplementary Information:**

The online version contains supplementary material available at 10.1186/s12931-021-01786-w.

## Introduction

Extremely low gestational age neonates (ELGANs), born at < 28 weeks gestation often receive supplemental oxygen for respiratory support, with or without mechanical ventilation, during which they experience frequent, brief arterial oxygen desaturations, or apneas, which is associated with bradycardia and intermittent hypoxia (IH). In particular, ELGANs (22–23 weeks gestation) begin ventilation at the canalicular–saccular stage of lung development, well before alveolar differentiation, and surfactant production [1]. Studies show that the duration of IH shorten but increase in severity during the first month of life, and the majority of IH occurs in clusters [2]. We have shown that clustered IH events are more damaging than dispersed IH events in neonatal rats [3]. Exposure of immature lungs to supraphysiological oxygen, combined with surfactant deficiency and poor nutrition result in poor alveolar development and microvascular maturation.

Fluctuations in oxygenation is a risk factor for long-term respiratory dysfunction, such as bronchopulmonary dysplasia [4, 5], a form of chronic lung disease (CLD) of prematurity [6, 7]. Hallmarks of the disease include vascular changes, infiltration with inflammatory cells, and alternating areas of alveolar over-inflation and atelectasis with pulmonary fibrosis [8]. Recent studies show that oxygen toxicity/hyperoxia and oxidative stress plays a significant role in the development of BPD/CLD [9]. Oxidative stress occurs when antioxidant defense mechanisms are insufficient to cope with reactive oxygen species (ROS) production, either through increased production of ROS or through insufficient presence of antioxidants. ELGANS are deficient in antioxidants which generally increase with advancing gestation, rendering them susceptible to oxidative stress and related diseases [10, 11]. Previously, studies have demonstrated an association between oxidative stress and BPD [12–14]. Coenzyme Q10 (CoQ10) is a powerful antioxidant involved in oxidative phosphorylation and is an essential part of the cellular machinery used to produce adenosine triphosphate (ATP). CoQ10 acts as a primary scavenger of free radicals, and is synthesized in-vivo [15]. Lipid emulsions high in omega 3 polyunsaturated fatty acids, such as fish oil, are widely used to improve growth and neurodevelopment in ELGANs. Fish oil supplementation has been shown to reduce oxidative stress in ELGANs [16]. Lipids are highly susceptible to reactive oxygen species (ROS) attack and lipid peroxidation [17].

We therefore tested the hypothesis that CoQ10 is more effective than fish oil for prevention of IH-induced lung injury in neonatal rats. Our hypothesis was tested with the following objectives: (1) To compare the effects of two clinically-relevant neonatal IH paradigms on lung antioxidants and biomarkers of oxidative stress; and (2) To determine whether supplementation with CoQ10 has better therapeutic outcomes than fish oil for preventing IH-induced lung injury. Our primary outcomes were lung histopathology and related morphometry, and our secondary outcomes were biomarkers of oxidative stress and antioxidants in the lungs.

## Materials and methods

### Animals

Certified infection-free, timed-pregnant Sprague Dawley rats were purchased from Charles River Laboratories (Wilmington, MA) at 18 days gestation. The animals were housed in an animal facility with a 12-h-day/12-h-night cycle and provided standard laboratory diet and water ad libitum until delivery of their pups.

### Experimental design

Within 2–4 h of birth, newborn rat pups delivering on the same day were pooled and randomly assigned to expanded litters of 18 pups/litter (nine males and nine females) for each experimental group (Additional file 1: Figure S1), as previously described [18–21]. Equal numbers for each gender in each group normalized gender differences. Expanded litter sizes in each group were implemented to simulate poor nutrition and growth experienced by ELGANs who are at the highest risk for oxidative lung injury. This is important to determine which treatment was more efficacious. Animals were exposed to neonatal IH from P0 to P14, or allowed to recover in room air (RA) until P21 with no further treatment. RA littermates, raised in atmospheric oxygen from P0 to P14 or P0 to P21, were similarly supplemented. We exposed rats to neonatal IH for 14 days because, similar to ELGANs who require oxygen therapy and who experience frequent IH episodes, rat lungs at birth are exclusively in the early saccular stage and bulk alveolarization occurs between P4-P13 [22, 23], and microvasculature is still immature. Animals were allowed to recover in room air (RA) for 1 week (P14) to determine reperfusion injury. At P21, bulk alveolarization is almost complete.

### Neonatal intermittent hypoxia (IH) profiles

Episodes of re-oxygenation following an IH event may occur in normoxia or hyperoxia. The two IH paradigms were: (1) exposure to 50% O_2_ with brief hypoxia (50%/12% O_2_ IH); or (2) exposure to normoxia with brief hypoxia (21%/12% O_2_ IH), as previously described [18]. Briefly, the IH profiles consisted of: (1) an initial exposure of hyperoxia (50% O_2_) for 30 min followed by three brief, 1-min, clustered hypoxic events (12% O_2_), with a 10-min re-oxygenation in 50% O_2_ between each hypoxic event. Recovery from IH occurred in 50% O_2_ following each clustered IH event for 2.5 h for a total of 8 clustering IH episodes per day for 14 days; or (2) an initial exposure of normoxia (21% O_2_) for 30 min followed by three brief, 1-min, clustered hypoxic events (12% O_2_), with a 10-min re-oxygenation in 21% O_2_ between each hypoxic event. Recovery occurred in normoxia following each clustered IH event for 2.5 h for a total of 8 clustering IH episodes per day for 14 days. These IH profiles are clinically relevant to those infants who experience IH with recovery in either hyperoxia or RA between episodes.

### Treatment

From P0-P14, pups (n = 18 pups per treatment group) were administered daily oral doses of: (1) CoQ10 (0.35 mg in 50 µL extra virgin olive oil) purchased from Sigma Aldrich (St. Louis, MO USA); (2) 50 µL fish oil containing 35 mg total *n*-3 PUFAs (22 mg eicosapentaenoic acid, EPA and 13 mg docosahexaenoic acid, DHA); or (3) 50 µL extra virgin olive oil. Treatments were administered using an orogastric syringe. The doses of CoQ10 and fish oil were based on our previous data [20, 21].

### Sample collection and processing

Pups were euthanized at P14 or allowed to recover from neonatal IH in RA from P14 to P21. Total body weight (grams) and linear growth (crown to rump length, cm) were recorded at P0, P14, and P21 to determine percentage change and weight accretion from birth. Weight accretion evidenced by percentage changes in body weight and linear growth were calculated as weight or length at the end of the experiment (P14 or P21) minus weight or length at birth (P0) divided by the weight or length at birth × 100. Percentage change in body weight was used to standardize differences in birth weight. Whole lungs were excised and weighed to determine lung/body weight ratios. Lung to body weight ratios appear to be a more important and accurate representation of the effects of the experimental compounds on organs [24]. Biopsies were collected, rinsed in ice-cold phosphate buffered saline (PBS, pH 7.4) on ice and placed in Lysing Matrix D 2.0 mL sterile tubes containing ceramic beads (MP Biomedicals, Santa Ana, CA, USA) for assay of oxidants and antioxidants.

### Lungs histology and morphometry

For histopathology and immunofluorescence (IF) staining, whole lungs (n = 6/experimental group) were infused in-situ with 10% neutral-buffered formalin (NBF), and fixed in 10% NBF for H&E staining. Unstained sections were used for IF staining. Lungs fixed in 10% NBF were processed, embedded, sectioned, and stained by the State University of New York (SUNY) Downstate Medical Center Pathology Department using standard laboratory techniques. Images were captured at 20× magnification (scale bar = 50 µm) using an Olympus BX53 microscope, DP72 digital camera, and CellSens imaging software (Olympus, Center Valley, PA), attached to a Dell Precision T3500 computer (Dell, Round Rock, TX). Morphometric analyses included total alveolar space (μM^2^); number of secondary crests; and width of the alveolar septae (μM) [25]. Ten sections were analyzed for each group. Measurements were made using the count and measure tool of the Olympus CellSens imaging software. Unstained sections were used for IF staining of SOD-1, SOD-2, SOD-3, and catalase.

### Assay of oxidants

Levels of 8-iso-prostaglandin F_2α_ (8-isoPGF_2α_), hydrogen peroxide (H_2_O_2_), and malondialdehyde (MDA) were determined in the lung homogenates using commercially available kits purchased from Enzo Life Sciences (Farmingdale, NY, USA, USA), Cayman Chemical Co. (Ann Arbor, MI, USA), and Millipore-Sigma (St. Louis, MO, USA), respectively. All samples were processed and assayed according to the manufacturer’s protocol. A total of 6 samples per experimental group were analyzed. All data were standardized using total cellular protein levels.

### Assay of antioxidants

Levels of SOD, catalase and total GSH, and total antioxidant capacity in the lung homogenates were determined using commercially available activity assay kits purchased from Cayman Chemical (Ann Arbor, MI, USA) and Millipore-Sigma (St. Louis, MO, USA), respectively. A total of 6 samples per experimental group were analyzed. Samples were processed and assayed according to the manufacturer’s protocol and standardized using total cellular protein levels.

### Total cellular protein levels

On the day of assays an aliquot (10 µL) of the lung homogenates was utilized for total cellular protein levels using the Bradford method (Bio-Rad, Hercules, CA USA) with bovine serum albumin as a standard.

### Immunofluorescence

Unstained lung cross-sections from 6 animals per experimental group were de-paraffinized with xylenes and alcohol prior to unmasking of antigens. There were two sections per slide for each of the 6 animals per experimental group. Sections were washed several times in PBS containing triton X-100 (PBS-T) and incubated in blocking solution (5% goat serum) for 1 h. The blocking serum was removed and primary antibodies for SOD-1, SOD-2, SOD-3, and catalase (Santa Cruz Biotechnology, Dallas, TX, USA) were added, prior to incubation overnight. Sections were incubated with Alexa Fluor fluorescent secondary antibodies (ThermoFisher Sci/Life Technologies, Grand Island, NY), counterstained with DAPI and images were captured at 20× magnification (scale bar = 50 µm) using an Olympus BX53 microscope, DP72 digital camera, and CellSens imaging software attached to a Dell Precision T3500 computer (Olympus America, Inc. USA). Quantitative analysis of the staining intensity was conducted using the count and measure on ROI tool of the CellSens software (*n* = 12 measurements/group).

### Statistical analysis

Differences among the RA and the two IH groups, and differences among the supplemented groups within each oxygen environment were analyzed using two-way analysis of variance (ANOVA) with Dunnett’s post-hoc tests, following the Bartlett’s test for normality. Kruskall Wallis nonparametric test with Dunn’s multiple comparison was used for non-normally distributed data. Data were analyzed using the Statistical Package for the Social Sciences (SPSS) software, version 26.0 (SPSS Inc., Chicago, IL, USA) and are reported as mean ± standard error of the mean (SEM). A *p*-value of < 0.05 was considered as statistically significant.

## Results

### Weights

Overall, exposure to both IH paradigms (control groups supplemented with olive oil) resulted in lower percentage change in body weight at P14 (50/12% O_2_: 225.5 ± 7.1%, p < 0.01; 21/12% O_2_: 191.8 ± 9.4%, p < 0.01) and remained suppressed during the recovery/reoxygenation period at P21 (50/12% O_2_: 319.9 ± 12.3%, p < 0.01; 21/12% O_2_: 327.2 ± 12.1%, p < 0.01) compared to RA at P14 (328.1 ± 7.5%) and P21 (513.0 ± 13.5%). However, the suppressive effects of IH on body weight accretion were reversed with fish oil at P21 (50/12% O_2_: 499.4 ± 19.3%; 21/12% O_2_: 484.8 ± 15.3%). Organ weight is one of the most sensitive indicators of toxicity due to treatment with an experimental compound, and weight differences between treated and control animals may still occur in the absence of any morphological changes. Mean lung weights and lung/body weight ratios are presented in Additional file 11: Table S1. In RA, both treatments resulted in lower lung weights at P21 compared to OO. In IH, only OO decreased lung weights at P21 compared to RA. Fish oil treatment in 21%/12% O_2_ IH increased lung weights, but decreased lung/body weight ratios at P21.

### Lung histopathology

**Fig. 1:**
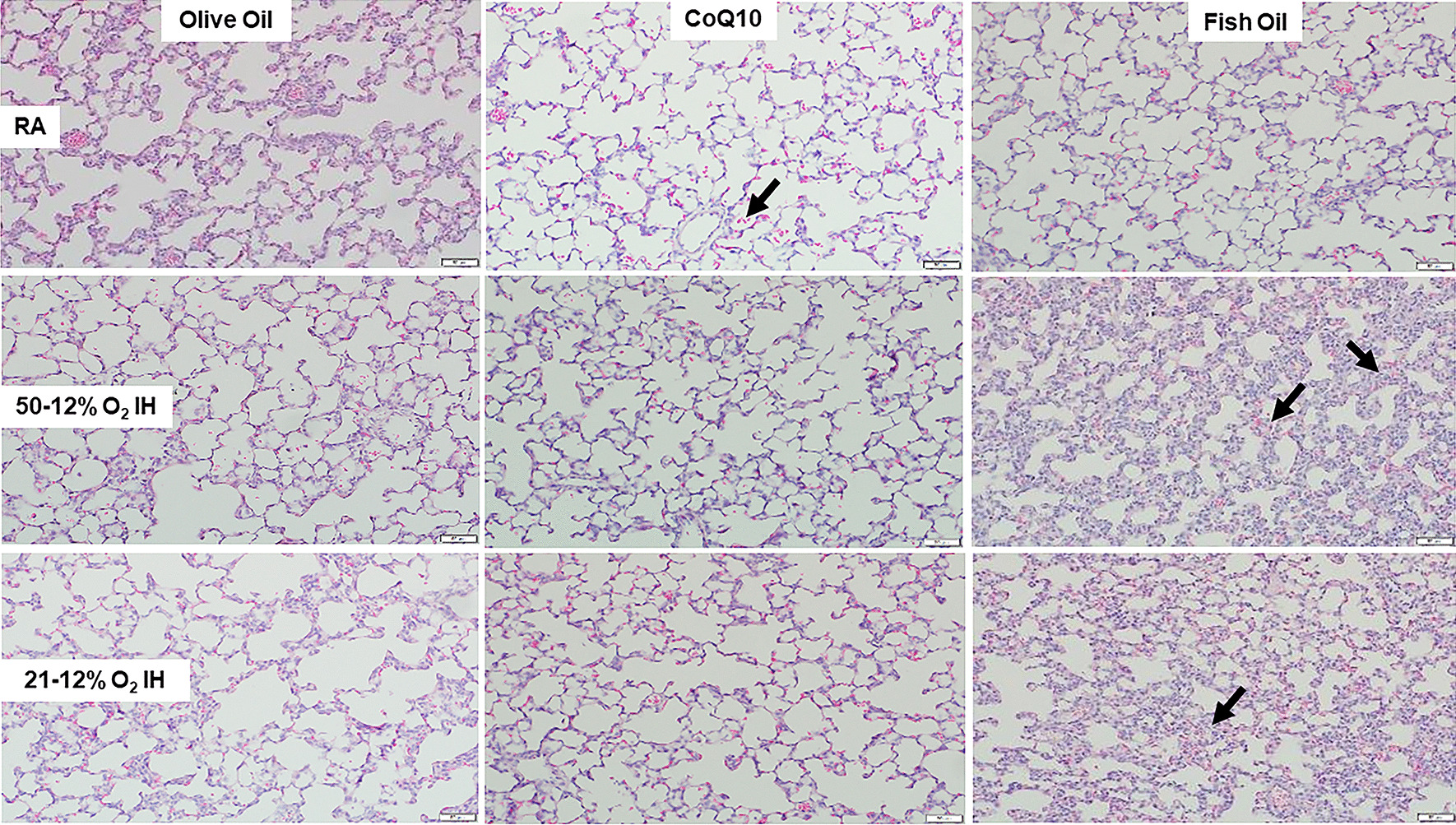
Representative H&E stained images of lungs from 14-day old rat exposed to room air (RA, top panels), 50%/12% O_2_ intermittent hypoxia (IH, middle panels), and 21%/12% O_2_ IH (lower panels). Animals were supplemented with olive oil (OO, left panels), coenzyme Q10 (CoQ10, middle panels), or fish oil (right panels). Images are 20× magnification and the scale bar is 50 µM. Arrows show hemorrhage and simplified alveoli. H&E images of lungs from non-supplemented rats at P14 and P21 exposed to neonatal IH are presented in Additional file 1: Figure S1

**Fig. 2:**
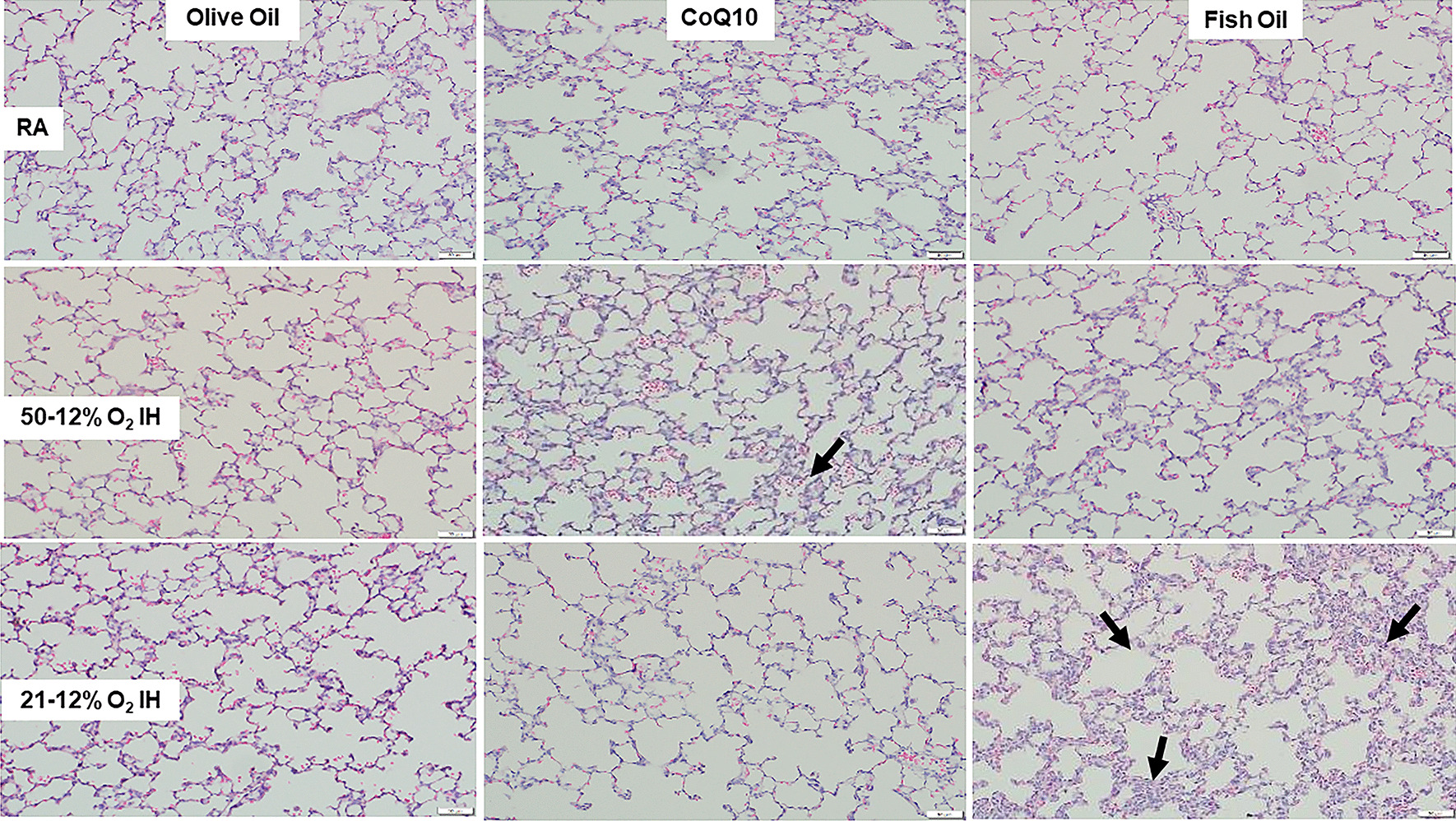
Representative H&E stained images of lungs from 21-day old rat exposed to room air (RA, top panels), 50%/12% O_2_ intermittent hypoxia (IH, middle panels), and 21%/12% O_2_ IH (lower panels). Animals were supplemented with olive oil (OO, left panels), coenzyme Q10 (CoQ10, middle panels), or omega 3 polyunsaturated fatty acids (fish oil, right panels). Images are 20× magnification and the scale bar is 50 µM. Arrows show hemorrhage and thickened septae

Representative H&E stains of lung tissue sections from rats at P14 and P21 are presented in Figs. 1 and 2, respectively (20× magnification, scale bar is 50 µm). At P14, treatment with OO in RA showed mild hemorrhage and alveoli with thickened septae and reduced secondary crest formation. CoQ10 and fish oil treatment in RA also showed mild hemorrhage, but alveoli were more mature. Treatment with OO and CoQ10 in 50%/12% O_2_ IH did not result in any major abnormalities, but treatment with fish oil showed immature, simplified alveoli with reduced secondary crest formation, thickened septae, and mild hemorrhage (arrows). These histopathological changes persisted in 21%/12% O_2_ IH. At P21, there were no major abnormalities seen in the RA groups, except for larger alveoli noted in the fish oil group. However, mild hemorrhage persisted in the groups exposed to both neonatal IH paradigms. Interestingly, lung damage persisted in the group treated with fish oil in 21%/12% O_2_ IH evidenced by oversimplified alveoli, reduced secondary crest formation, thickened septae, and mild hemorrhage (arrows). H&E stained lung tissue sections from rats at P14 and P21 that were exposed to neonatal IH but were untreated, are presented in Additional file 2: Figure S2 (20× magnification, scale bar is 50 µm). The upper panel represents the groups exposed to 50%/12% O_2_ IH, and the middle and lower panels represent the groups exposed to 21%/12% O_2_ IH. Exposure to 51%/12% O_2_ resulted in mild hemorrhage with reduced alveoli at P14 (arrows). At P14, while evidence of hemorrhage persisted, the alveoli were larger. In contrast, animals exposed to 21%/12% O_2_ IH showed pervasive hemorrhage at P14 with simplified alveoli, loss of secondary crests, and thickened septae which persisted at P21 (arrows).

### Lung morphometry

Quantitative morphometric analyses for lungs at P14 and P21 are presented in Additional file 12: Table S2, Additional file 13: Table S3, respectively. At P14, both treatment groups exposed to room air showed a decrease in septal thickness when compared to OO controls. In IH, CoQ10 supplementation decreased alveolar number, length of secondary crests, and alveolar diameter and area, but increased the number of secondary crests, compared to OO. In contrast, fish oil treatment in IH decreased all parameters, except septal thickness which was elevated in 50%/12% O_2_ IH. At P21, the effects persisted particularly with fish oil treatment in 21%/12% O_2_ IH.

### Oxidative stress

**Fig. 3:**
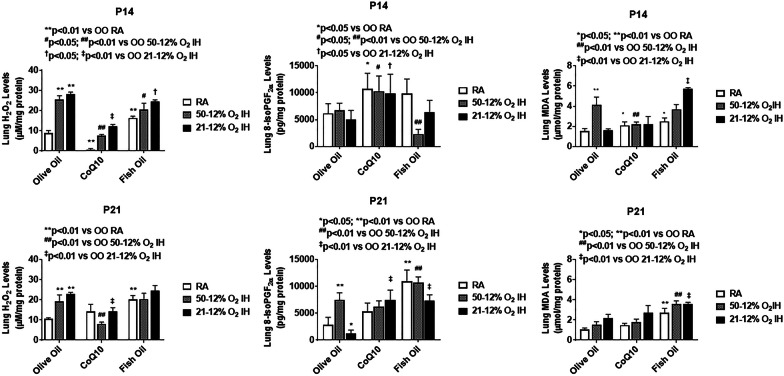
Effects of CoQ10 or fish oil supplementation during neonatal IH on the oxidants, hydrogen peroxide (H_2_O_2_), 8-isoprostaglandin F_2α_ (8-isoPGF_2α_), and malondialdehyde (MDA), in lung homogenates on postnatal day 14 (P14) and P21. Pups were exposed to neonatal IH from P0 to P14 during which they were supplemented with CoQ10 or fish oil. Post IH, animals were either euthanized at P14 or placed in RA with no further supplementation for re-oxygenation/recovery until P21. RA control littermates (white bar) remained in RA from birth to P14 or P21. The shaded bars represent groups exposed to 50%/12% O_2_ IH, and the solid black bars represent groups exposed to 21%/12% O_2_ IH. Data are expressed as mean ± SEM (*n* = 6 samples/group)

**Fig. 4:**
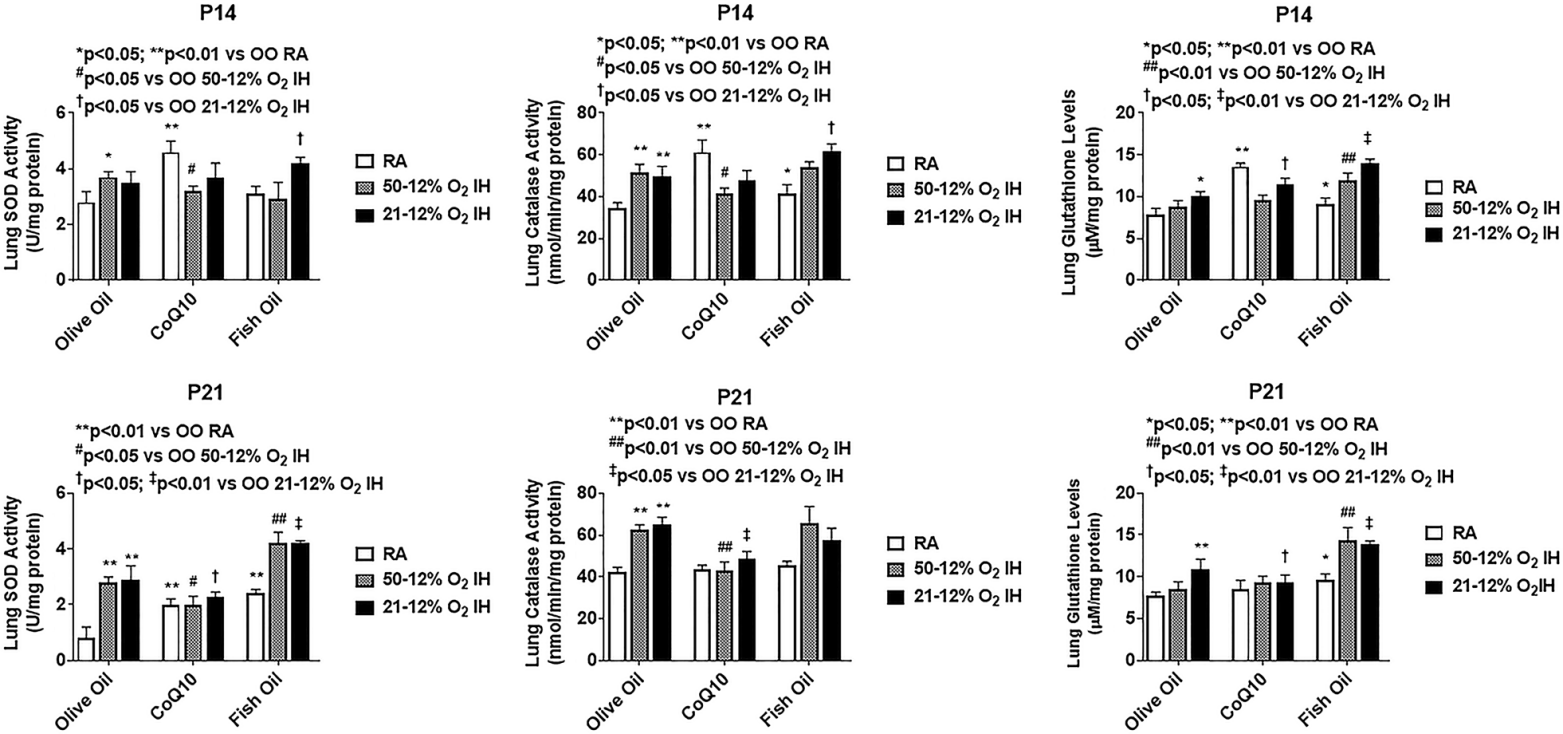
Effects of fish oil or CoQ10 supplementation during neonatal IH on the antioxidants, superoxide dismutase (SOD), catalase or glutathione, in lung homogenates on postnatal day 14 (P14) and P21. Pups were exposed to neonatal IH from P0 to P14 during which they were supplemented with fish oil or COQ10. Groups are as described in Fig. 1. Data are expressed as mean ± SEM (*n* = 6 samples/group

Levels of oxidants (H_2_O_2_, 8-isoPGF_2α_, and MDA) in the lung homogenates at P14 and P21, are presented in Fig. 3. H_2_O_2_ and 8-isoPGF_2α_ levels in the lung homogenates are presented in Fig. 4. At P14, both neonatal IH paradigms significantly increased lung H_2_O_2_ levels. CoQ10 effectively suppressed H_2_O_2_ in all oxygen environments, but fish oil was less effective and resulted in elevated H_2_O_2_ levels in RA. This effect persisted at P21, although a rebound elevation of H_2_O_2_ was seen with CoQ10 treatment in RA. A quite different effect was noted for 8-isoPGF_2α_. CoQ10 caused significant elevations in 8-isoPGF_2α_ levels in all oxygen conditions at P14 and fish oil reduced it in 50%/12% O_2_ IH. At P21, 8-isoPGF_2α_ levels remained elevated in 50%/12% O_2_ IH with all treatments, but the highest levels occurred with fish oil. In 21%/12% O_2_ IH, 8-isoPGF_2α_ levels increased with both CoQ10 and fish oil. Lung MDA, representing lipid peroxidation, was elevated with olive oil in 50%/12% O_2_ IH, and with fish oil in IH. At P21, sustained MDA increases were seen with in fish oil in all oxygen conditions.

### Antioxidants

**Fig. 5:**
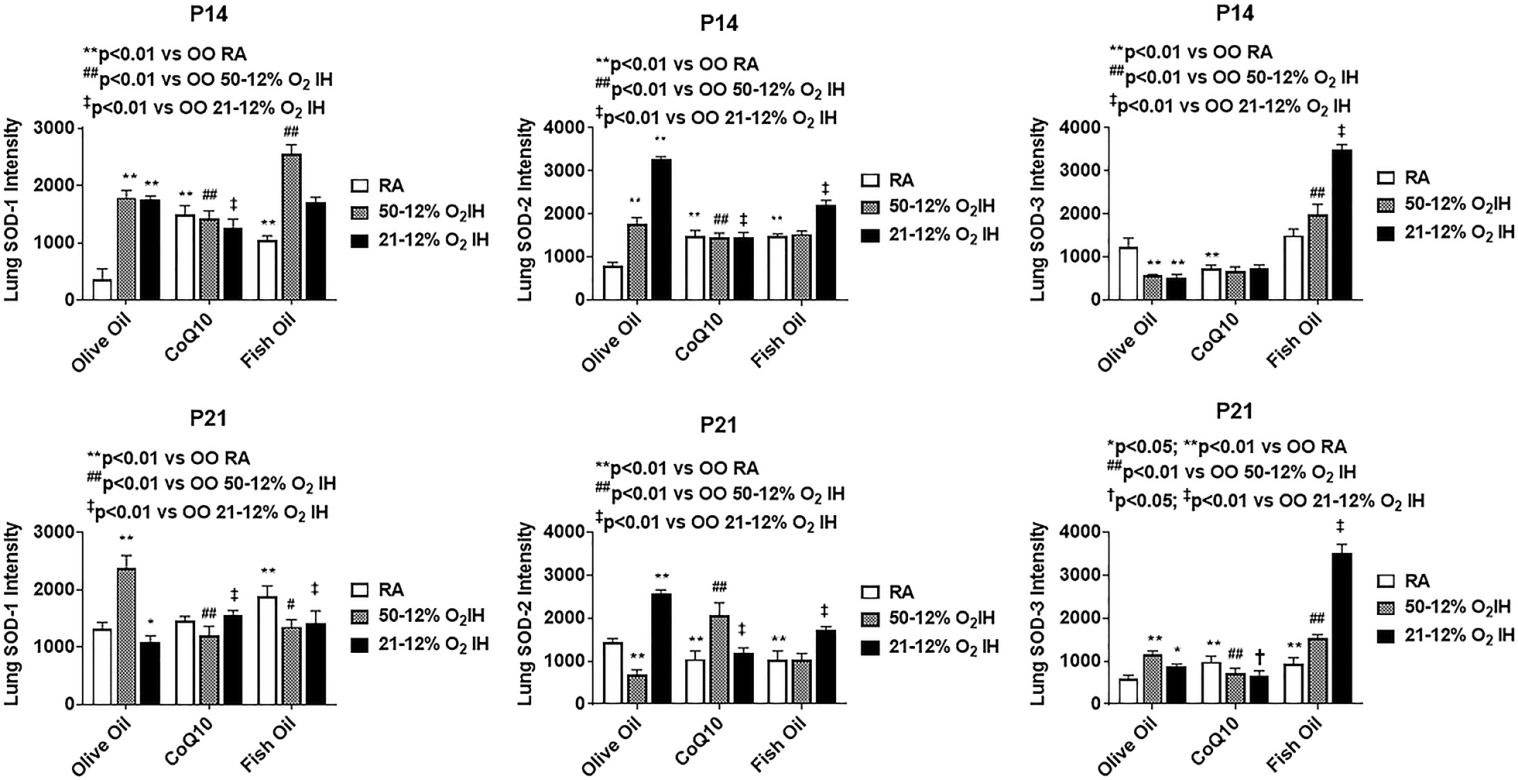
Quantitative analysis of SOD-1, SOD-2, and SOD-3 immunofluorescence staining intensity in the lung sections from rats at P14 and P21. Corresponding images are presented in the Additional file 2: Figure S2, Additional file 3: Figure S3, Additional file 4: Figure S4, Additional file 5: Figure S5, Additional file 6: Figure S6, Additional file 7: Figure S7). Groups are as described in Fig. 1. Data are presented as mean ± SEM (*n* = 12 samples/group)

**Fig. 6:**
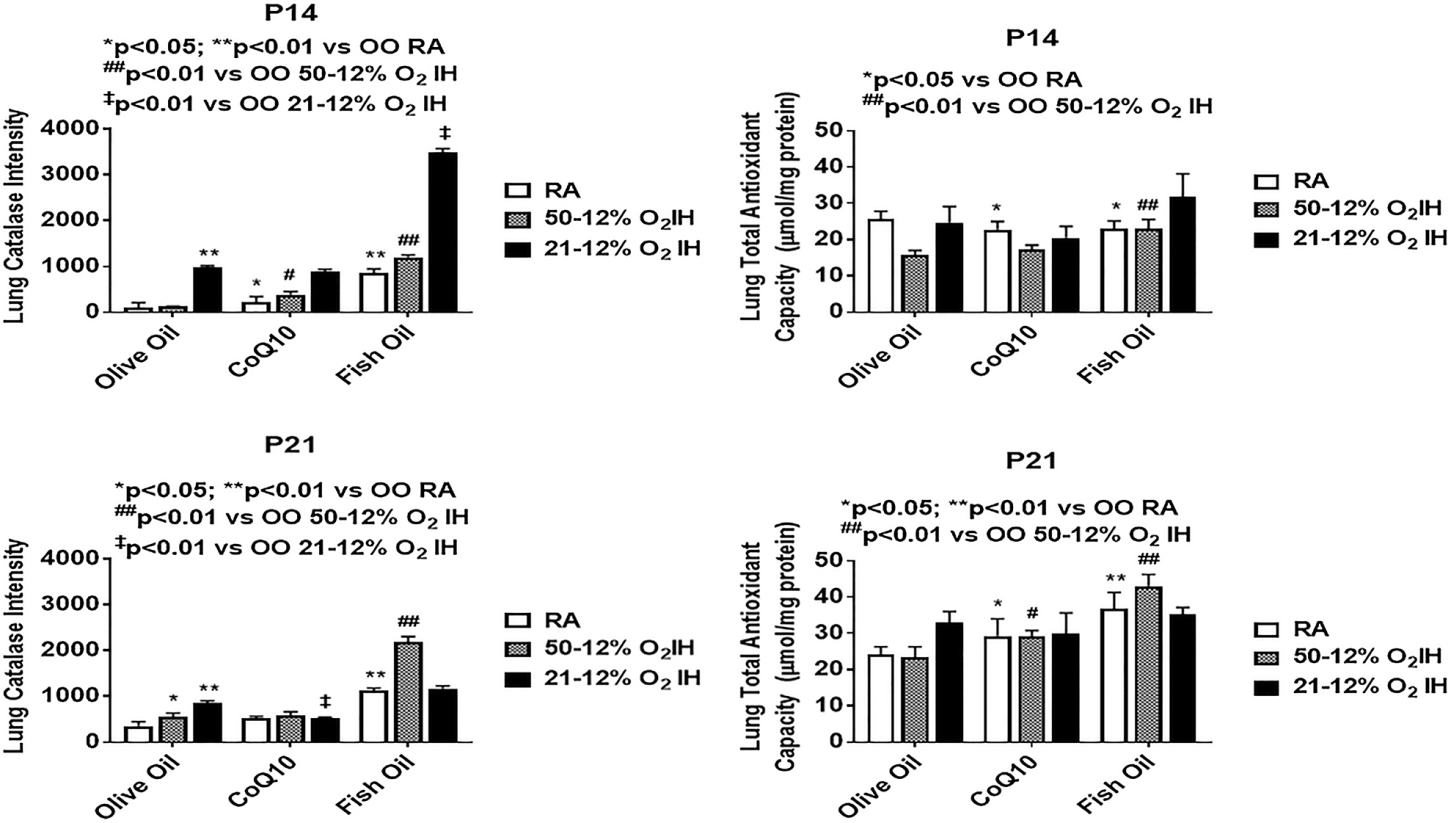
Quantitative analysis of catalase immunofluorescence staining intensity in the lung sections, and total antioxidant capacity in lung homogenates, from rats at P14 and P21. Corresponding catalase images are presented in the Additional file 8: Figure S8, Additional file 9: Figure S9. Groups are as described in Fig. 1. Data are presented as mean ± SEM (*n* = 12 samples/group for catalase intensity and *n* = 6 samples/group for total antioxidant capacity)

Levels of antioxidants and total antioxidant capacity in the lung homogenates are presented in Figs. 2, 3, 4, 5, 6. Total SOD, catalase and glutathione at P14 and P21 is presented in Fig. 4. At P14, SOD was significantly elevated with CoQ10 in RA, and OO in 50%/12% O_2_ IH compared to OO treatment in RA. Catalase activity was induced in the OO and fish oil groups exposed to IH, but lowered with CoQ10. Total glutathione levels were higher with fish oil treatment in both IH conditions, but with OO and CoQ10 in 21%/12% O_2_ IH only. At P21, SOD and catalase activities were elevated in response to both IH paradigms with OO and fish oil supplementation. Glutathione was increased with OO in 21%/12% O_2_ IH group and with fish oil treatment in both IH conditions. Corresponding IF stains of SOD-1, SOD-2, SOD-3, and catalase at P14 and P21 are shown in Additional file 3: Figure S3, Additional file 4: Figure S4, Additional file 5: Figure S5, Additional file 6: Figure S6, Additional file 7: Figure S7, Additional file 8: Figure S8, Additional file 9: Figure S9, Additional file 10: Figure S10, respectively. Quantitative analyses of the IF stains for SOD isoforms in the lung sections are presented in Fig. 5. At P14, SOD-1 was elevated with all treatments compared to OO in RA, but was higher with fish oil. SOD-2 was higher with OO in both IH conditions, and with fish oil in 21%/12% O_2_ IH. SOD-3 was increased with fish oil in all oxygen environments. At P21, the effects on SOD-2 and SOD-3 persisted with OO and fish oil. Quantitative analysis of catalase IF stains in the lung sections, and total antioxidant capacity in the lung homogenates at P14 and P21, are presented in Fig. 6. At P14, catalase increased with all treatments in 21%/12% O_2_ IH, but the effect was substantial with fish oil. Total antioxidant capacity was higher with OO and fish oil in 21%/12% O_2_ IH. At P21, elevations in catalase and total antioxidant capacity persisted in the fish oil group.

## Discussion

The major findings of this report are: (1) while both neonatal IH paradigms were damaging to the immature lungs, resolution with normoxia between IH episodes was more injurious than IH resolving in hyperoxia. This finding was unexpected, and is likely due to the shorter FiO_2_ range from 21 to 12% compared to 50% to 12%, which allowed the lungs more respite from the effects of hypoxia; (2) CoQ10 was more effective for reducing IH-induced lung injury than fish oil, and the damage was associated with significant elevations in H_2_O_2_ and MDA. Both H_2_O_2_ and MDA are indicators of lipid peroxidation. H_2_O_2_ is produced by dismutation of superoxide anion by SOD and in excess, can react with free iron to form the reactive hydroxyl radical which destroys lipids, proteins, and DNA via the Fenton-Haber–Weiss reaction [26]. MDA is formed as a product of lipid peroxidation, the reaction of oxygen with unsaturated lipids, and appears to be the most mutagenic product of lipid peroxidation [27]; and (3) reduced lung/body weight ratios in the control groups exposed to neonatal IH may be reflective of overall abnormal growth and development of the lungs which persisted during the recovery/reoxygenation period. These findings strongly imply that in the setting of neonatal IH, the use of lipids may perpetuate oxidative stress injury due to its susceptibility to oxidation. Although frequently described as having antioxidant properties, studies show that fish oil are highly prone to oxidation [28]. Therefore, combining lipids with antioxidants may improve its therapeutic efficacy and reduce the effects of ROS on lipids, and may be essential for preserving its stability and therapeutic efficacy in neonatal IH [29]. Further studies are needed to confirm this.

The two IH paradigms used in this study were designed to simulate the acute oxygen desaturations seen in ELGANs with respiratory distress syndrome. Both paradigms address the clinical question of whether resolution of an IH episode with room air or hyperoxia between episodes is more beneficial to the developing lungs. In our study, we found that lung damage worsened during recovery/reoxygenation at P21, and was in line with many studies which reported that hypoxia/reoxygenation events produce the most damaging outcomes, known as reperfusion injury, of which oxygen free radicals appear to be highly involved [30]. Comparing the two IH paradigms, it is likely that 21%/12% O_2_ IH model may be associated with a higher activation of hypoxia/ischemia. Reoxygenation in 21% O_2_ between episodes was insufficient to counteract the effects, before the next IH insult initiated. Restitution of blood flow during reperfusion injury occurs in damaged, leaky vessels leading to hemorrhage which was noted predominantly with the 21/12% O_2_ IH paradigm. On the other hand, reoxygenation in 50% O_2_ following neonatal IH provided a higher FiO_2_ that may be necessary to override the mechanisms associated with hypoxia/ischemia. Although both paradigms are deleterious to the developing lungs, increasing oxygen for resolution between episodes may reduce the severity of lung damage.

In oxidative stress, the first oxidant produced is superoxide anion. The natural defense against superoxide is its dismutation by SOD to H_2_O_2_ and oxygen. Subsequently, H_2_O_2_ is detoxified by catalase and glutathione. These antioxidants are induced or repressed to match the intensity of ROS production. Fish oil supplementation in IH caused robust production of oxidants and responsive antioxidants. This finding strongly suggests that in the setting of neonatal IH, the antioxidant benefits of fish oil may be diminished. Specifically, fish oil is consumed in the diet and absorbed via the gut, and have been implicated as a trigger for oxidative stress and inflammation in the intestine [31]. Recent studies showed that fish oil, at best, does not decrease the incidence of BPD [32, 33]. The finding of elevated antioxidants during recovery/reoxygenation in the fish oil group strongly suggest a robust response to increased ROS production. Whether increased ROS is due to fish oil peroxidation, diminished antioxidant capacity, or both remains to be determined. CoQ10 was shown to effectively suppress oxidative damage and oxidants in the lungs exposed to both neonatal IH paradigms. CoQ10 also did not affect overall lung weight or lung to body weight ratios. More importantly, CoQ10 was associated with lower SOD and GSH which are generally induced in response to oxidative stress and may indicate a necessary mitochondrial defense [34]. In the lung SOD-3 is the predominant isoform and has been implicated as a major player in the prevention of hyperoxic lung injury and BPD [35–37]. Notably, robust elevations occurred in the fish oil groups, particularly with exposure to 21%/12% O_2_ IH which also manifested the most damage, and produced characteristics consistent with BPD [38]. Under normal conditions, after day 10 of life, the lung of the neonatal rat should show signs of further septation, including lengthening and thinning of old septa and development of secondary septa to form new alveoli [22]. CoQ10 supplementation was shown to improve lung architecture despite exposure to neonatal IH. However, further studies are needed to determine its safety and efficacy for use in preterm infants.

## Conclusions

Lipids emulsions are widely used in neonates to improve growth, visual function, and neurodevelopmental outcomes. The efficacy of lipids for prevention of lung inflammation is controversial. Our findings show that neonatal IH induces H_2_O_2_ and MDA, further supporting lipid peroxidation. CoQ10, remains promising in the prevention of a multitude of human diseases. However, its safety and efficacy for prevention and/or treatment of oxidative stress diseases of the neonate require appropriate and extensive preclinical investigations.

## Supplementary Information

**Additional file 1:** **Figure S1.** Flow chart of Experimental Design. Animals were placed in neonatal intermittent hypoxia (IH) conditions from the first day of life, postnatal day 0 (P0) until P14 during which the received oral supplementation with olive oil (control), coenzyme Q10, or fish oil. At P14, pups were placed in room air conditions until P21 for reoxygenation/reperfusion, with no further supplementation. Room air littermates remained in normoxic conditions from P0 to P21 and were similarly supplemented.

**Additional file 2: Figure S2.** Representative H&E stained images of lungs from non-supplemented rats exposed to 50%/12% O2 intermittent hypoxia (IH) or 21%/12% O2 IH at P14 and P21. Images are 20× magnification and the scale bar is 50 µM. Arrows show hemorrhage and simplified alveoli.

**Additional file 3: Figure S3.** Representative image showing immunoreactivity of superoxide dismutase (SOD)-1 in the lung sections from groups supplemented with fish oil or CoQ10 during neonatal IH at P14. Images are 20× magnification and the scale bars are 50 µM.

**Additional file 4: Figure S4.** Representative image showing immunoreactivity of superoxide dismutase (SOD)-1 in the lung sections from groups supplemented with fish oil or CoQ10 during neonatal IH at P21. Images are 20× magnification and the scale bars are 50 µM.

**Additional file 5: Figure S5. **Representative image showing immunoreactivity of superoxide dismutase (SOD)-2 in the lung sections from groups supplemented with fish oil or CoQ10 during neonatal IH at P14. Images are 20× magnification and the scale bars are 50 µM.

**Additional file 6: Figure S6. **Representative image showing immunoreactivity of superoxide dismutase (SOD)-2 in the lung sections from groups supplemented with fish oil or CoQ10 during neonatal IH at P21. Images are 20× magnification and the scale bars are 50 µM.

**Additional file 7: Figure S7. **Representative image showing immunoreactivity of superoxide dismutase (SOD)-3 in the lung sections from groups supplemented with fish oil or CoQ10 during neonatal IH at P14. Images are 20× magnification and the scale bars are 50 µM.

**Additional file 8: Figure S8. **Representative image showing immunoreactivity of superoxide dismutase (SOD)-3 in the lung sections from groups supplemented with fish oil or CoQ10 during neonatal IH at P21. Images are 20× magnification and the scale bars are 50 µM.

**Additional file 9: Figure S9.** Representative image showing immunoreactivity of catalase in the lung sections from groups supplemented with fish oil or CoQ10 during neonatal IH at P14. Images are 20× magnification and the scale bars are 50 µM.

**Additional file 10: Figure S10.** Representative image showing immunoreactivity of catalase in the lung sections from groups supplemented with fish oil or CoQ10 during neonatal IH at P21. Images are 20× magnification and the scale bars are 50 µM.

**Additional file 11: Table S1.** Lung Weights and Lung/Body Weight Ratios.

**Additional file 12: Table S2**: Lung Morphometric Analyses at P14.

**Additional file 13: Table S3**: Lung Morphometric Analyses at P21.

## Data Availability

The datasets used and/or analyzed during the current study are available from the corresponding author on reasonable request.
