# Supplementary figures and images for: Comparison of coenzyme Q10 or fish oil for prevention of intermittent hypoxia-induced oxidative injury in neonatal rat lungs
Source: Respir Res. 2021 Jul 5;22:196. doi: 10.1186/s12931-021-01786-w (PMC8256540; doi:10.1186/s12931-021-01786-w)

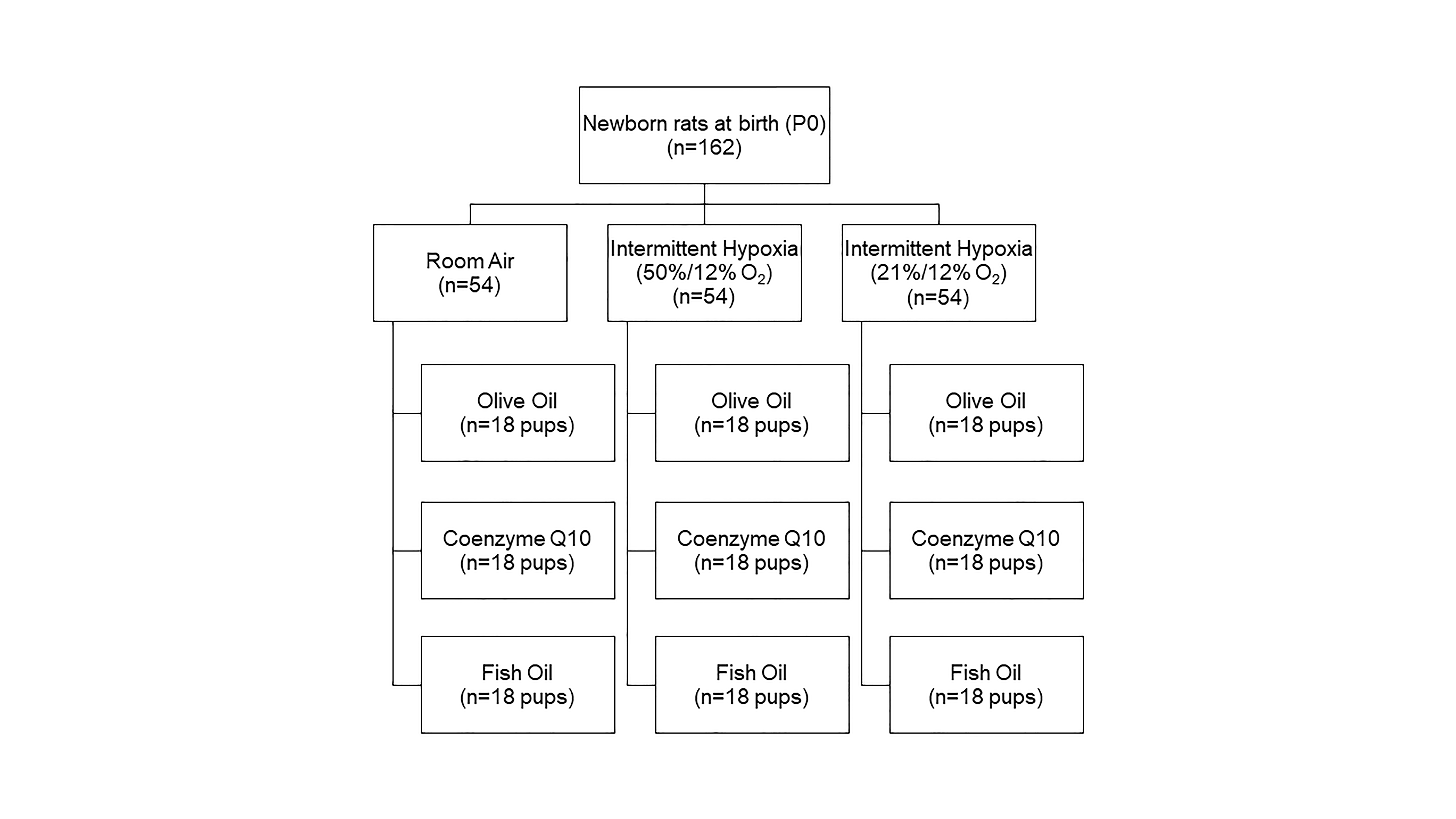

Supplement: Supplementary file 1 — Additional file 1: Figure S1. Flow chart of Experimental Design. Animals were placed in neonatal intermittent hypoxia (IH) conditions from the first day of life, postnatal day 0 (P0) until P14 during which the received oral supplementation with olive oil (control), coenzyme Q10, or fish oil. At P14, pups were placed in room air conditions until P21 for reoxygenation/reperfusion, with no further supplementation. Room air littermates remained in normoxic conditions from P0 to P21 and were similarly supplemented. [file 12931_2021_1786_MOESM1_ESM.tif]

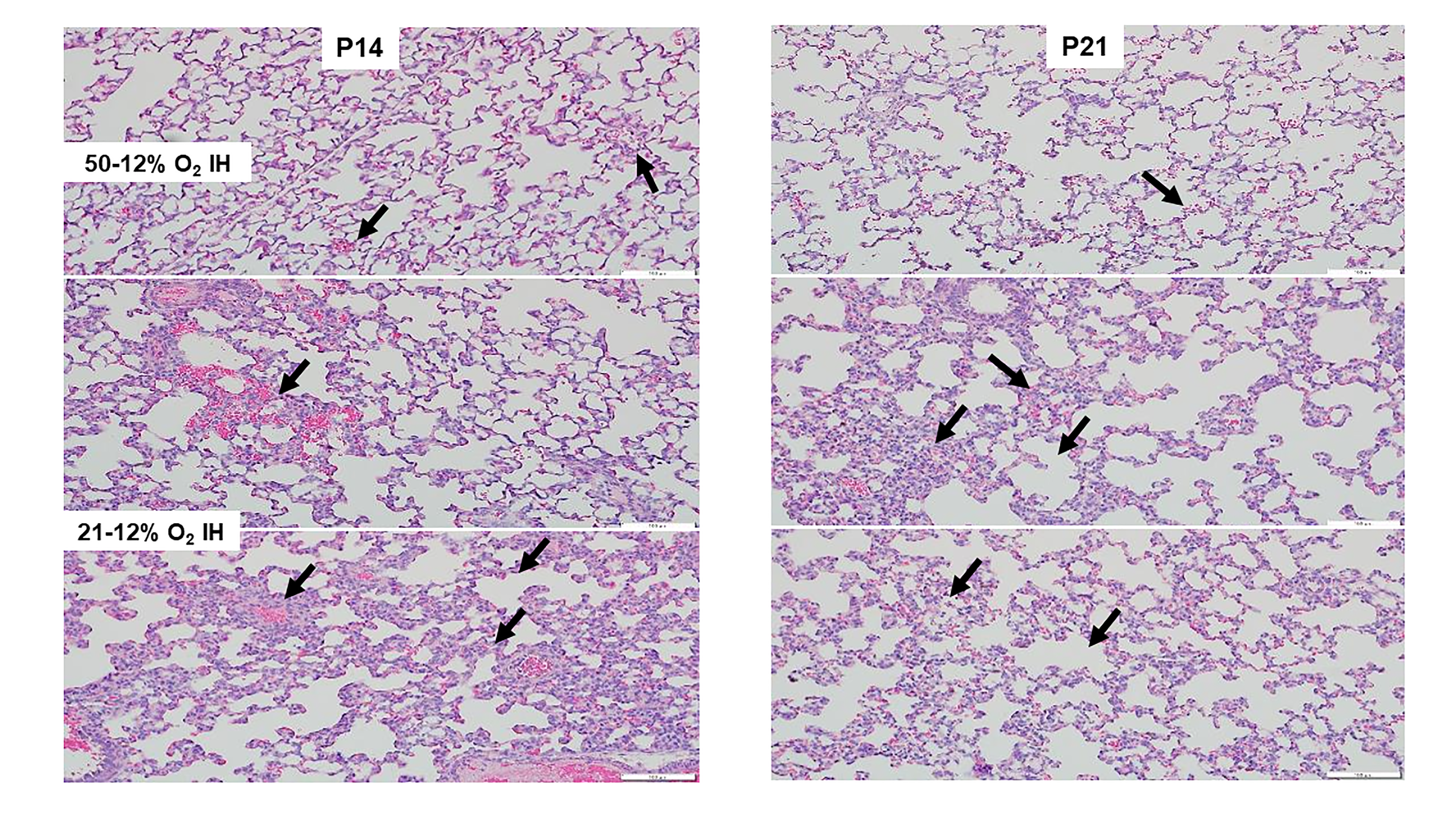

Supplement: Supplementary file 2 — Additional file 2: Figure S2. Representative H&E stained images of lungs from non-supplemented rats exposed to 50%/12% O2 intermittent hypoxia (IH) or 21%/12% O2 IH at P14 and P21. Images are 20× magnification and the scale bar is 50 µM. Arrows show hemorrhage and simplified alveoli. [file 12931_2021_1786_MOESM2_ESM.tif]

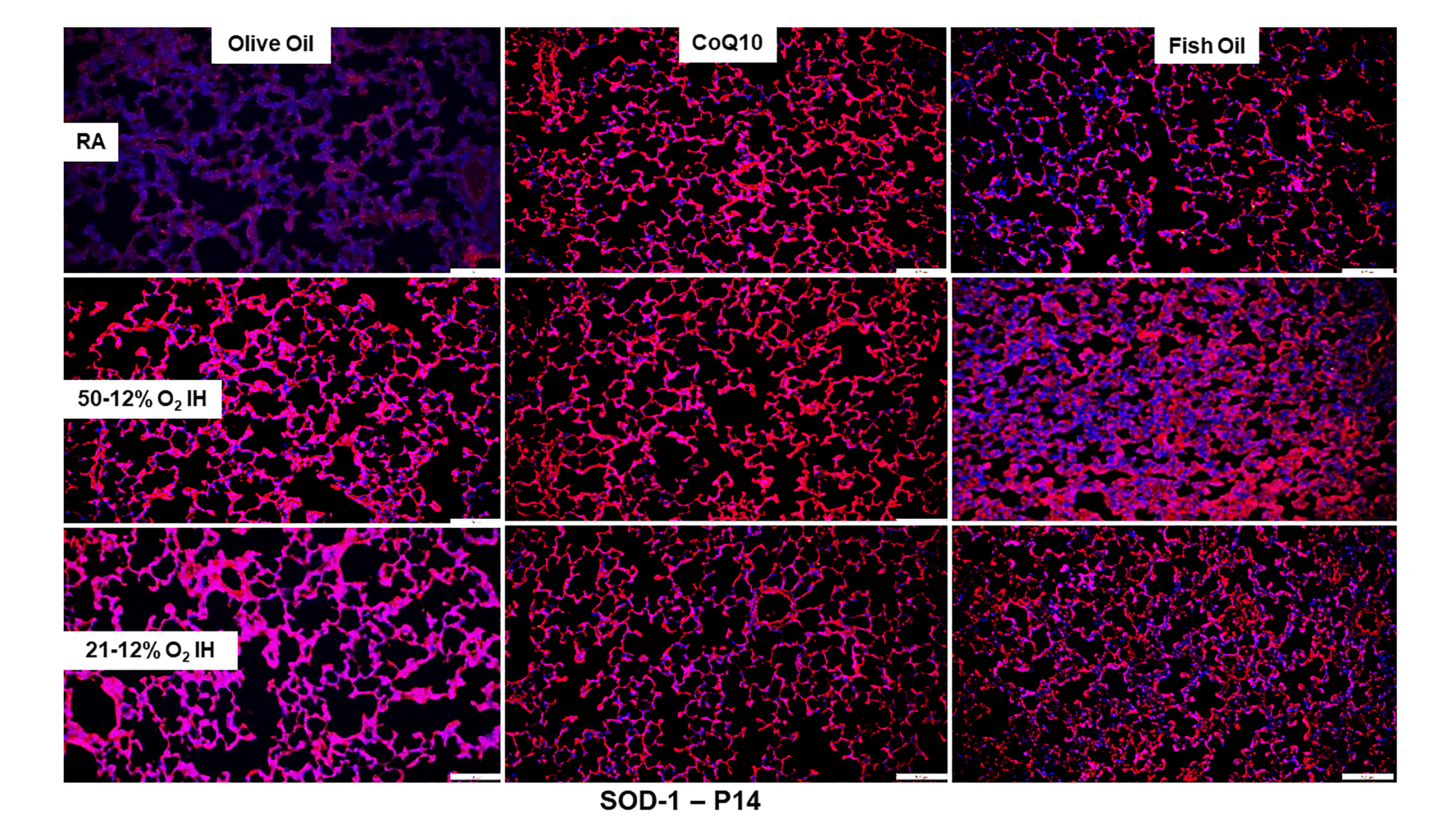

Supplement: Supplementary file 3 — Additional file 3: Figure S3. Representative image showing immunoreactivity of superoxide dismutase (SOD)-1 in the lung sections from groups supplemented with fish oil or CoQ10 during neonatal IH at P14. Images are 20× magnification and the scale bars are 50 µM. [file 12931_2021_1786_MOESM3_ESM.tif]

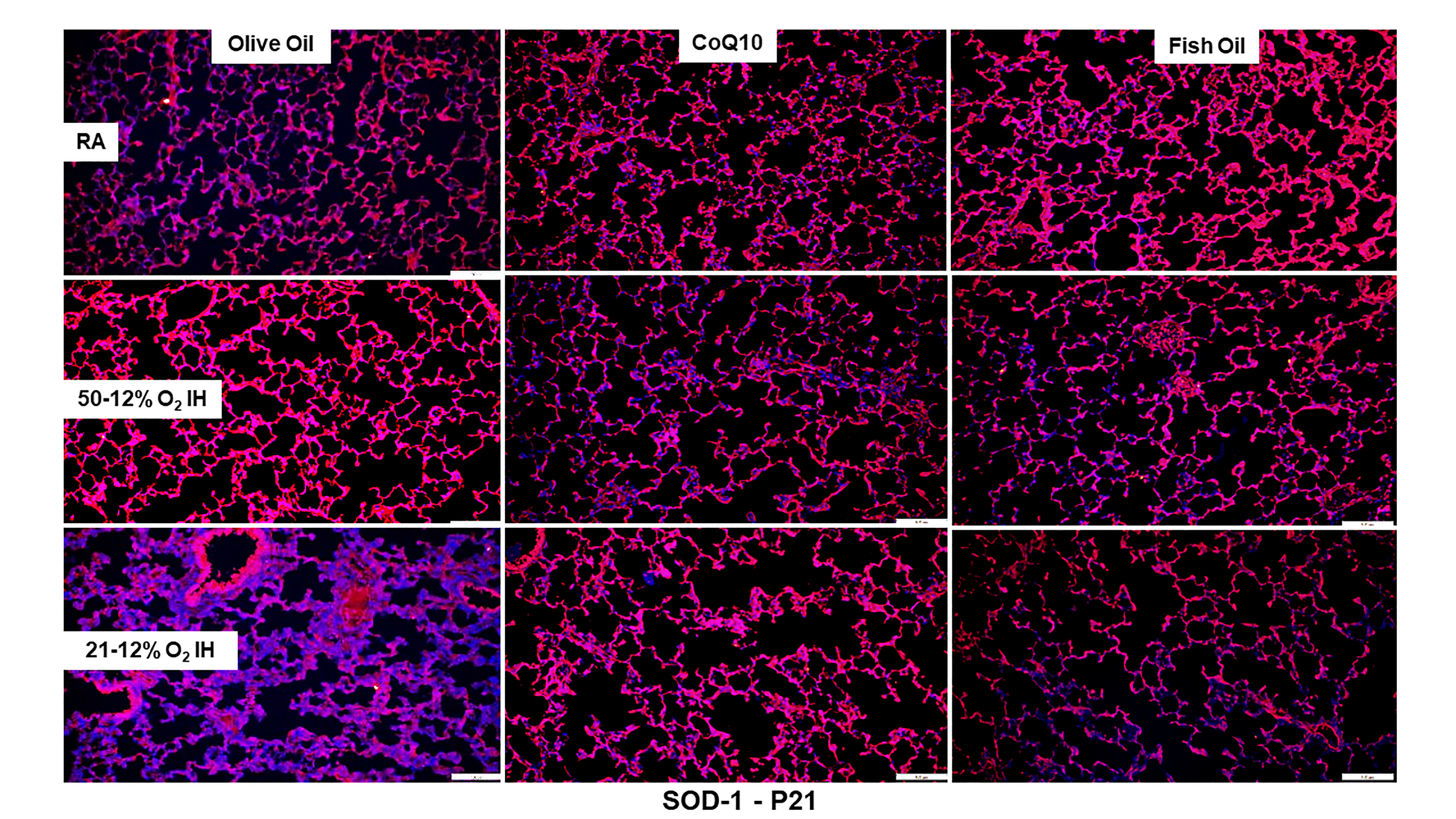

Supplement: Supplementary file 4 — Additional file 4: Figure S4. Representative image showing immunoreactivity of superoxide dismutase (SOD)-1 in the lung sections from groups supplemented with fish oil or CoQ10 during neonatal IH at P21. Images are 20× magnification and the scale bars are 50 µM. [file 12931_2021_1786_MOESM4_ESM.tif]

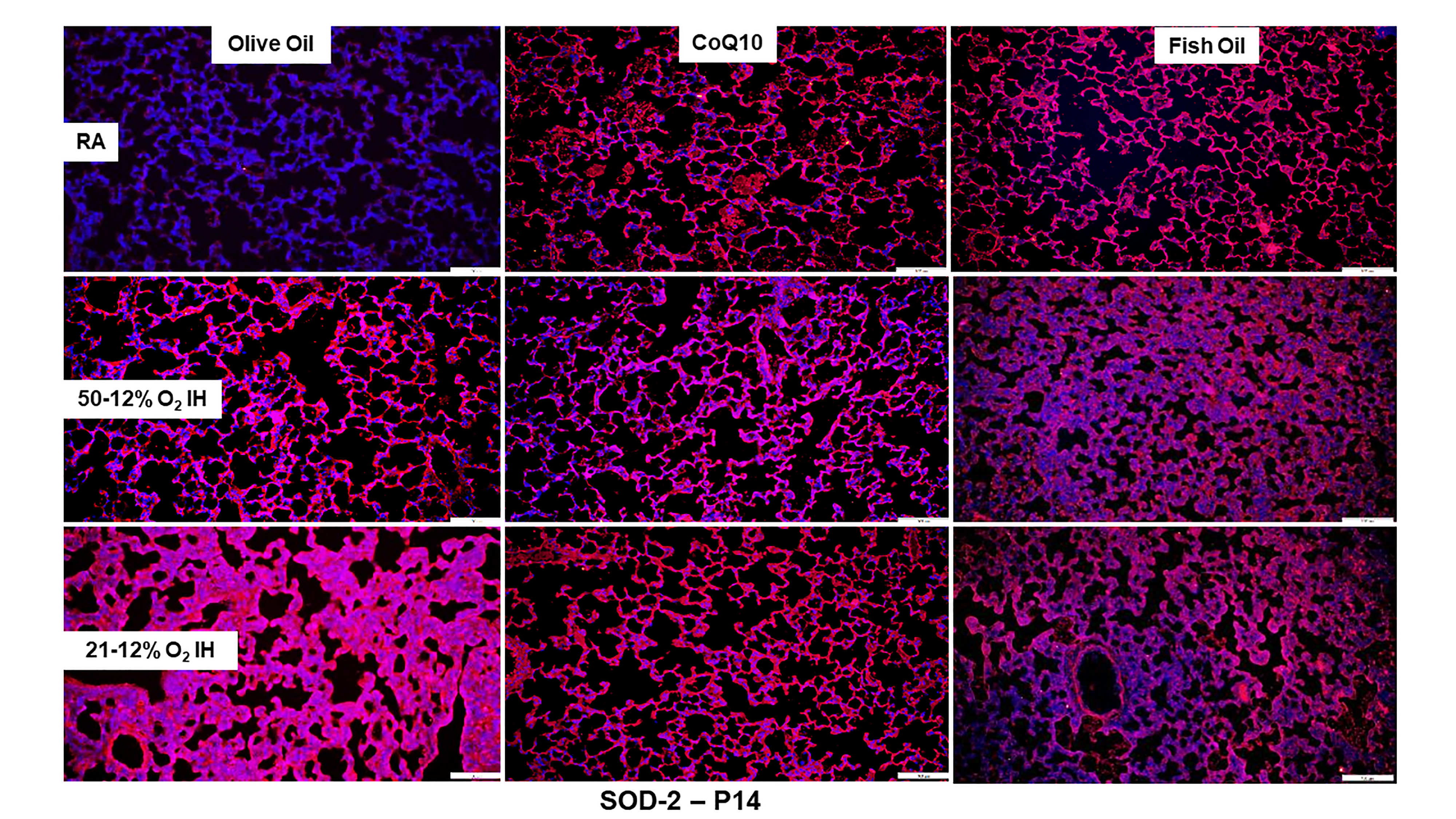

Supplement: Supplementary file 5 — Additional file 5: Figure S5. Representative image showing immunoreactivity of superoxide dismutase (SOD)-2 in the lung sections from groups supplemented with fish oil or CoQ10 during neonatal IH at P14. Images are 20× magnification and the scale bars are 50 µM. [file 12931_2021_1786_MOESM5_ESM.tif]

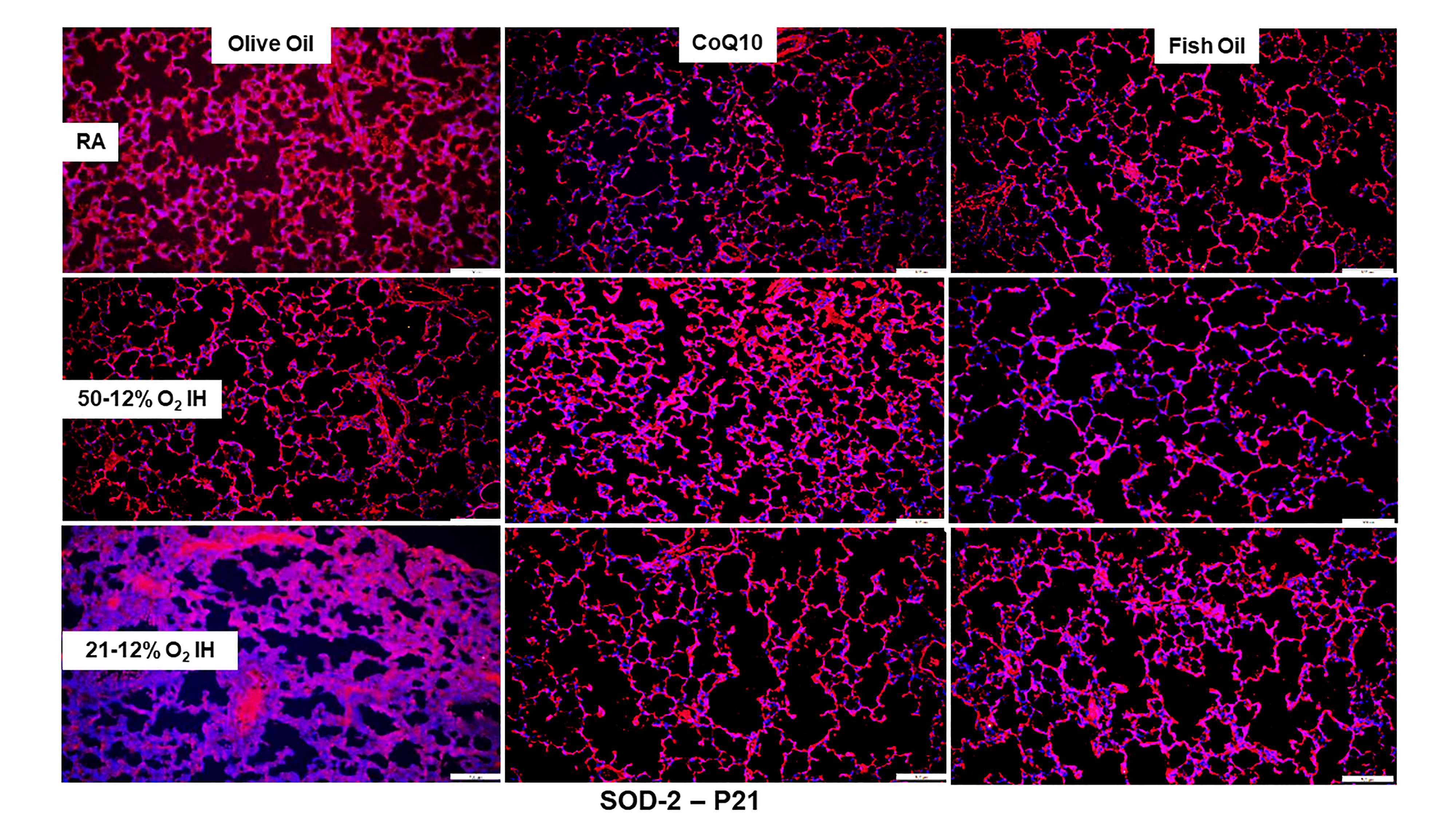

Supplement: Supplementary file 6 — Additional file 6: Figure S6. Representative image showing immunoreactivity of superoxide dismutase (SOD)-2 in the lung sections from groups supplemented with fish oil or CoQ10 during neonatal IH at P21. Images are 20× magnification and the scale bars are 50 µM. [file 12931_2021_1786_MOESM6_ESM.tif]

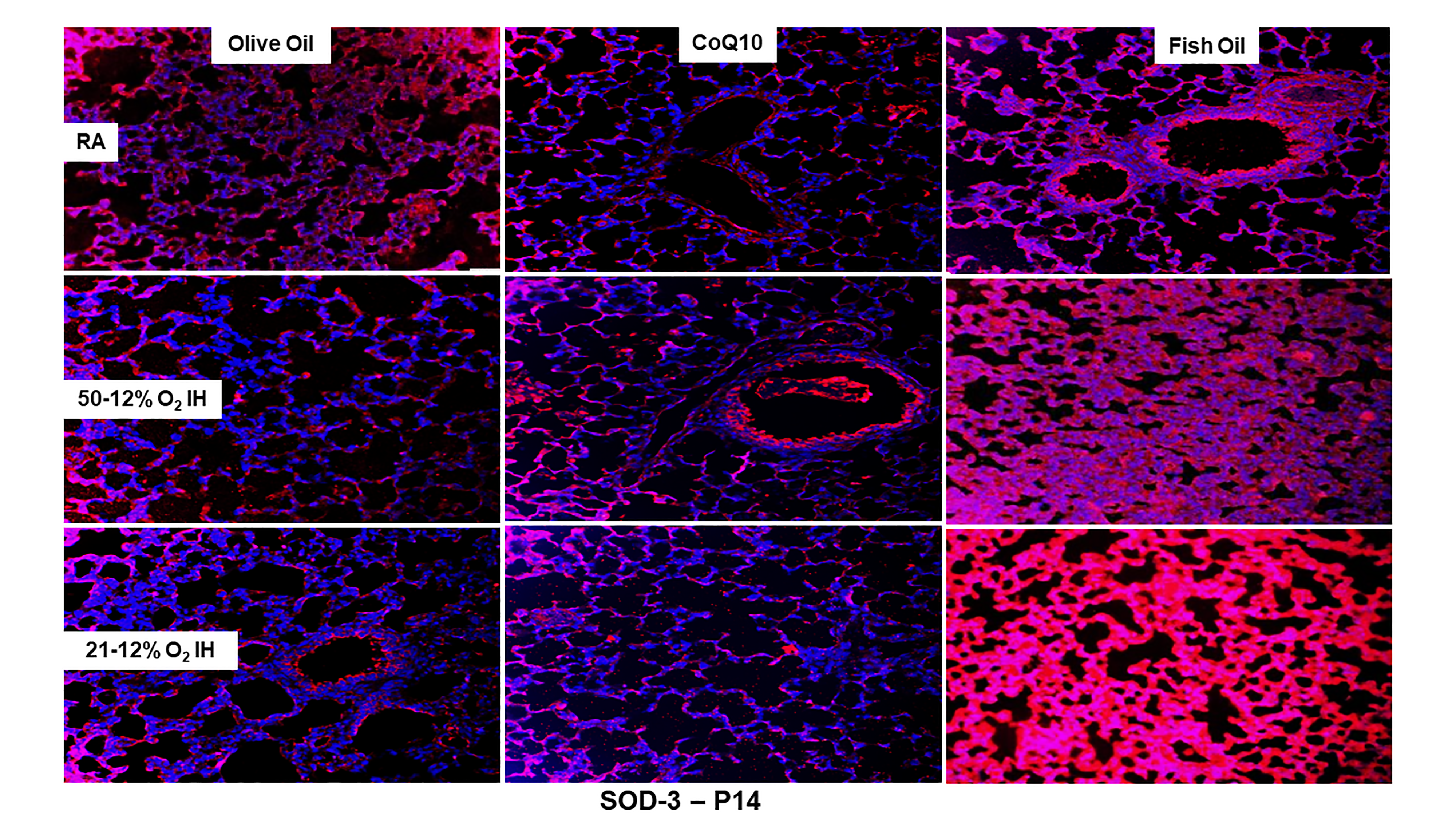

Supplement: Supplementary file 7 — Additional file 7: Figure S7. Representative image showing immunoreactivity of superoxide dismutase (SOD)-3 in the lung sections from groups supplemented with fish oil or CoQ10 during neonatal IH at P14. Images are 20× magnification and the scale bars are 50 µM. [file 12931_2021_1786_MOESM7_ESM.tif]

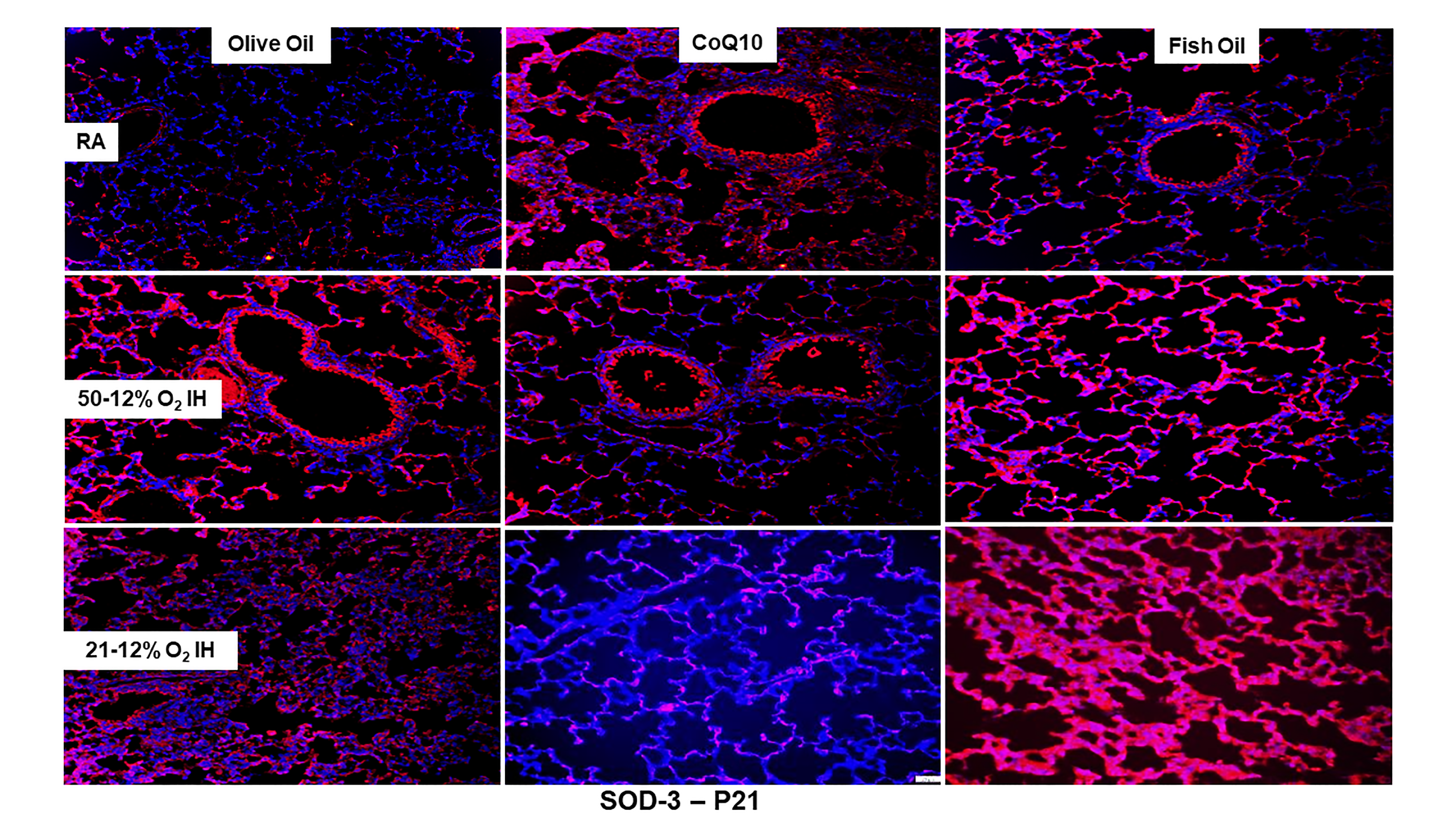

Supplement: Supplementary file 8 — Additional file 8: Figure S8. Representative image showing immunoreactivity of superoxide dismutase (SOD)-3 in the lung sections from groups supplemented with fish oil or CoQ10 during neonatal IH at P21. Images are 20× magnification and the scale bars are 50 µM. [file 12931_2021_1786_MOESM8_ESM.tif]

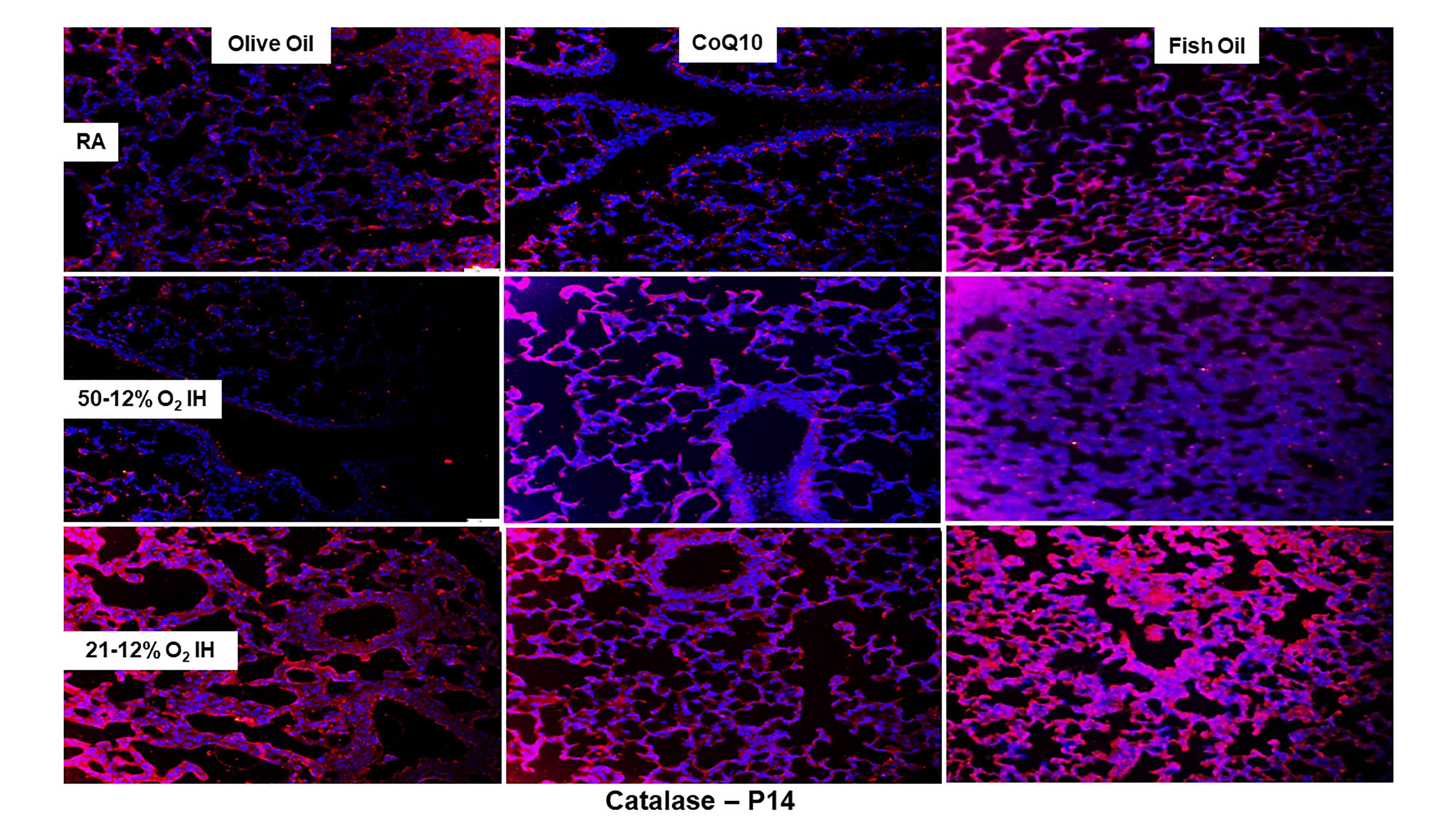

Supplement: Supplementary file 9 — Additional file 9: Figure S9. Representative image showing immunoreactivity of catalase in the lung sections from groups supplemented with fish oil or CoQ10 during neonatal IH at P14. Images are 20× magnification and the scale bars are 50 µM. [file 12931_2021_1786_MOESM9_ESM.tif]

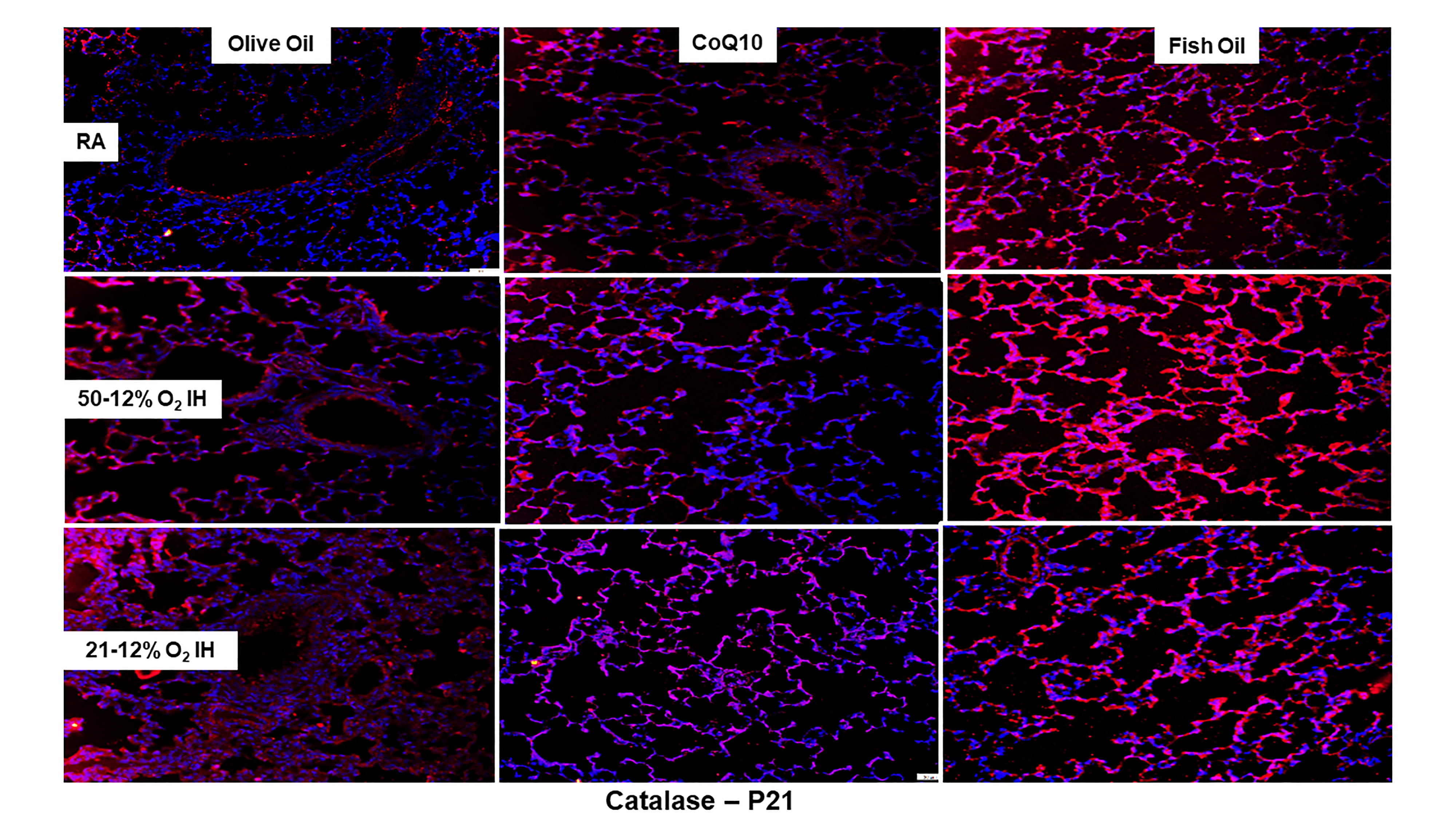

Supplement: Supplementary file 10 — Additional file 10: Figure S10. Representative image showing immunoreactivity of catalase in the lung sections from groups supplemented with fish oil or CoQ10 during neonatal IH at P21. Images are 20× magnification and the scale bars are 50 µM. [file 12931_2021_1786_MOESM10_ESM.tif]
