# Supplementary material for: Comparison of coenzyme Q10 or fish oil for prevention of intermittent hypoxia-induced oxidative injury in neonatal rat lungs
Source: Respir Res. 2021 Jul 5;22:196. doi: 10.1186/s12931-021-01786-w (PMC8256540; doi:10.1186/s12931-021-01786-w)
Supplement: Supplementary file 11 — Additional file 11: Table S1. Lung Weights and Lung/Body Weight Ratios. [file 12931_2021_1786_MOESM11_ESM.docx]

| **Groups** | **Lung Wt. at P14** | **Lung/Body Wt. Ratio at P14** | **Lung Wt. at P21** | **Lung/Body Wt. Ratio at P21** |
| --- | --- | --- | --- | --- |
| ***Room Air (RA):*** | | | | |
| Olive Oil | 0.43±0.01 | 0.02±0.001 | 0.49±0.02 | 0.01±0.0004 |
| CoQ10 | 0.44±0.02 | 0.02±0.001 | 0.46±0.01# | 0.01±0.0003 |
| Fish Oil | 0.39±0.02 | 0.019±0.001 | 0.44±0.02# | 0.01±0.0006 |
| ***50%O_2_/12% O_2_ IH:*** | | | | |
| Olive Oil | 0.48±0.01 | 0.02±0.0008 | 0.35±0.01** | 0.01±0.0001 |
| CoQ10 | 0.39±0.01# | 0.02±0.0004 | 0.38±0.01 | 0.01±0.0004 |
| Fish Oil | 0.48±0.01 | 0.02±0.0004 | 0.45±0.01 | 0.01±0.0001 |
| ***21%O_2_/12% O_2_ IH:*** | | | | |
| Olive Oil | 0.41±0.02 | 0.02±0.001 | 0.33±0.02** | 0.01±0.0007 |
| CoQ10 | 0.43±0.02 | 0.02±0.001 | 0.36±0.004 | 0.01±0.0003 |
| Fish Oil | 0.41±0.01 | 0.019±0.001 | 0.41±0.02## | 0.009±0.0004# |

**Table 1:** Lung Weights and Lung/Body Weight Ratios

Data are mean±SD. Data were analyzed using two-way ANOVA. *p<0.05, **p<0.01 vs RA; ^#^p<0.05, ^##^p<0.01 vs Olive Oil (n=18 pups/group).
