# Supplementary material for: Comparison of coenzyme Q10 or fish oil for prevention of intermittent hypoxia-induced oxidative injury in neonatal rat lungs
Source: Respir Res. 2021 Jul 5;22:196. doi: 10.1186/s12931-021-01786-w (PMC8256540; doi:10.1186/s12931-021-01786-w)
Supplement: Supplementary file 12 — Additional file 12: Table S2: Lung Morphometric Analyses at P14. [file 12931_2021_1786_MOESM12_ESM.docx]

| **Groups** | **No. Alveoli**  **(n=4)** | **Thickness of Septae (µm)**  **(n=16)** | **No. Secondary crests (µm) (n=4)** | **Length of Secondary crests (n=42)** | **Alveolar diameter**  **(n=16)** | **Alveolar Area (µm^2^)**  **(n=16)** | **Alveolar Perimeter (µm)**  **(n=16)** | **Hemorrhage score**  **(n=4)** |
| --- | --- | --- | --- | --- | --- | --- | --- | --- |
| ***RA:*** | | | | | | | | |
| Olive Oil | 24±3.2 | 8.4±3.2 | 10.8±1.9 | 16.0±6.2 | 58.8±22.7 | 2103.9±1865.8 | 1331.1±2457.2 | 0 |
| CoQ10 | 23.3±2.8 | 5.0±1.2**##** | 15.5±3.0 | 15.3±6.0 | 46.4±17.7 | 1416.7±798.8 | 161.9±50.8**#** | 3 |
| Fish Oil | 21.0±4.3 | 6.2±1.3**##** | 12.0±3.4 | 12.7±5.1 | 50.2±12.7 | 1620.7±858.5 | 177.3±63.9**#** | 0 |
| ***IH (50%/12% O_2_):*** | | | | | | | | |
| Olive Oil | 24.8±3.0 | 7.0±2.1 | 8.8±2.5 | 21.7±12.0 | 56.1±14.5 | 2101.1±880.4 | 196.6±53.9 | 1 |
| CoQ10 | 19.8±2.2**#** | 6.7±1.6** | 12.8±2.2**#** | 16.0±6.9**##** | 43.5±12.5**#** | 1244.1±788.0**#** | 155.4±58.9 | 0 |
| Fish Oil | 15.0±2.2**##** | 11.4±2.0****##** | 1.3±0.96****##** | 4.3±7.5****##** | 49.5±17.2 | 1581.5±894.2 | 196.0±92.7 | 1.5 |
| ***IH (21%/12% O_2_):*** | | | | | | | | |
| Olive Oil | 15.0±6.5* | 10.2±5.0**#** | 12.3±1.3 | 18.0±7.1 | 81.5±22.6** | 3930.8±1924.1** | 300.1±116.2 | 0.75 |
| CoQ10 | 21.0±2.8 | 7.0±1.9** | 12.3±2.5 | 12.3±5.6**##** | 54.4±12.8**##** | 1842.1±871.2**##** | 188.0±50.9**##** | 1 |
| Fish Oil | 15.8±3.9 | 10.2±1.7** | 4.8±0.96****##** | 14.2±6.8**#** | 45.5±8.6**##** | 1555.0±775.7**##** | 175.4±55.4**##** | 2 |

**Supplemental Table 2**: Lung Morphometric Analyses at P14

Data are mean±SD. *p<0.05, **p<0.01 vs RA; ^#^p<0.05, ^##^p<0.01 vs Olive Oil (two-way ANOVA).
