# Supplementary material for: Comparison of coenzyme Q10 or fish oil for prevention of intermittent hypoxia-induced oxidative injury in neonatal rat lungs
Source: Respir Res. 2021 Jul 5;22:196. doi: 10.1186/s12931-021-01786-w (PMC8256540; doi:10.1186/s12931-021-01786-w)
Supplement: Supplementary file 13 — Additional file 13: Table S3: Lung Morphometric Analyses at P21. [file 12931_2021_1786_MOESM13_ESM.docx]

| **Groups** | **No. Alveoli**  **(n=4)** | **Thickness of Septae (µm)**  **(n=16)** | **No. Secondary crests (µm) (n=4)** | **Length of Secondary crests (n=42)** | **Alveolar diameter**  **(n=16)** | **Alveolar Area (µm^2^)**  **(n=16)** | **Alveolar Perimeter (µm)**  **(n=16)** | **Hemorrhage score**  **(n=4)** |
| --- | --- | --- | --- | --- | --- | --- | --- | --- |
| ***RA:*** | | | | | | | | |
| Olive Oil | 19.5±3.7 | 6.2±1.9 | 12.5±1.3 | 18.4±6.2 | 63.8±24.4 | 2612.1±1868.7 | 237.8±92.3 | 0 |
| CoQ10 | 20.5±3.1 | 6.9±1.5 | 9.0±3.5 | 17.4±6.1 | 48.0±14.2 | 1581.2±1201.2 | 162.4±60.9 | 1 |
| Fish Oil | 14.0±4.2 | 6.1±1.1 | 11.5±1.3 | 16.3±6.1 | 52.9±21.6 | 2225.6±2186.1 | 202.8±119.6 | 0 |
| ***IH (50%/12% O_2_):*** | | | | | | | | |
| Olive Oil | 17.5±3.0 | 6.3±2.4 | 12.0±1.8 | 15.1±6.4 | 57.9±24.0 | 2463.1±1909.4 | 212.8±93.9 | 0.5 |
| CoQ10 | 17.8±4.8 | 9.2±2.0****##** | 9.5±1.9 | 14.4±5.6* | 47.2±15.4 | 1528.7±889.7 | 167.8±55.8 | 1.5 |
| Fish Oil | 18.0±2.9 | 8.3±1.6****##** | 9.8±1.9 | 16.0±15.9 | 62.6±19.9 | 2526.8±1551.5 | 228.0±94.9 | 1 |
| ***IH (21%/12% O_2_):*** | | | | | | | | |
| Olive Oil | 15.3±3.1 | 8.7±4.3* | 11.8±4.1 | 19.4±8.4 | 73.9±27.6 | 3105.2±1949.9 | 257.0±91.8 | 1.75 |
| CoQ10 | 13.8±1.7* | 8.7±1.9* | 10.5±2.4 | 14.3±5.3***##** | 59.1±20.1 | 2336.7±1425.9 | 213.7±70.9* | 0.75 |
| Fish Oil | 13.8±2.6 | 11.3±2.3**# | 6.3±0.96****#** | 15.3±6.4**#** | 40.8±15.2**##** | 1366.8±898.1**##** | 152.5±55.6**##** | 2 |

**Supplemental Table 3**: Lung Morphometric Analyses at P21

Data are mean±SD. *p<0.05, **p<0.01 vs RA; ^#^p<0.05, ^##^p<0.01 vs Olive Oil (two-way ANOVA).
